# AGL-UNet: Adaptive Global–Local Modulated U-Net for Multitask Sea Ice Mapping

**DOI:** 10.3390/s26030959

**Published:** 2026-02-02

**Authors:** Deyang Chen, Fuqiang Zheng

**Affiliations:** 1 School of Information Science and Technology, Shanghai Tech University, Shanghai 201210, China; chendy2023@shanghaitech.edu.cn; 2 Shanghai Institute of Technical Physics, Chinese Academy of Sciences, Shanghai 200083, China; 3 Key Laboratory of Infrared System Detection and Imaging Technology, Shanghai Institute of Technical Physics, Chinese Academy of Sciences, Shanghai 200083, China; 4 Innovation Center for Fengyun Meteorological Satellite, Beijing 100081, China

**Keywords:** sea ice mapping, adaptive loss weighting strategy, multitask, multi-sensor data, convolutional neural network

## Abstract

The increasing demand for Arctic route planning, climate change studies, and the growing volume of satellite sensor data have made automated sea ice mapping an essential task. In this study, we propose a multi-task sea ice mapping framework based on the U-Net architecture, which supports multi-sensor data integration and automatically modulates global and local features. The model consists of ARC blocks for enhanced multi-sensor feature fusion, a GLCM block for non-local and local feature modulation, and an adaptive loss weighting strategy to balance multi-task training. The proposed method is evaluated on the AI4Arctic RTT dataset, which includes multi-sensor inputs and ice chart-derived labels. Compared with the best-performing method in the AutoIce Challenge, the proposed approach achieves a 1.33% improvement in the combined score. In addition, the F1 scores for stage of development (SOD) and floe size (FLOE) increase by 2.85% and 3.44%, respectively. Although the $R^{2}$ score for SIC shows a slight decrease of 1.25%, this behavior is consistent with the practical trade-offs commonly observed in multi-task optimization. Ablation studies further demonstrate the effectiveness of the proposed blocks and the multi-task adaptive weighting strategy, confirming their potential for handling multi-sensor data and supporting ocean environment monitoring.

## 1. Introduction

As a critical component of the Earth’s cryosphere, sea ice plays a vital role in regulating the global climate system, sustaining polar ecosystems, and supporting human activities in high-latitude regions [1,2]. Recent satellite observations show a continued decline in Arctic sea ice extent, which strengthens surface albedo feedback and accelerates polar warming far beyond the global average [3]. This environmental shift has also highlighted the strategic and economic significance of Arctic shipping routes, which may become key pathways in future global maritime transport [4]. Therefore, obtaining sea ice information is crucial for both global climate change research and Arctic shipping route planning. However, the vastness, remoteness, and rapidly evolving characteristics of the polar environment pose major challenges for traditional manual ice charting, which is labor intensive, time consuming, and difficult to apply on a large scale. In comparison, satellite remote sensing provides observations that are available in all weather conditions, at high temporal frequency, and over wide spatial coverage, forming the foundation of modern automated sea ice monitoring systems [5]. The expanding availability of multimodal satellite data has further enabled the development of advanced deep learning methods, which have significantly improved sea ice type classification, concentration estimation, and floe size analysis. These advances are essential for enhancing the realism of climate and sea ice models, improving the safety of polar navigation, supporting operational ice services, and deepening understanding of sea ice, ocean, and atmosphere interactions [6].

Over the past few decades, ice charts have served as a mainstream product for describing sea ice conditions by providing a range of sea ice parameters. However, these ice chart products require manual processes such as visual inspection and analysis of satellite imagery, which are highly inefficient and time-consuming. This underscores the necessity for automated sea ice mapping to enhance efficiency [7]. Deep learning has emerged as the primary approach in the field of automated sea ice mapping, aiding in the acquisition of sea ice parameters. Neural networks have been applied to sea ice mapping, such as [8,9,10] for estimating sea ice concentration (SIC), [11,12,13] for evaluating sea ice stage of development (SOD), and [14,15,16] for evaluating floe size (FLOE). These networks can automatically learn features from massive datasets, ensuring robust and precise estimates of sea ice parameters.

However, several limitations remain in existing automated sea ice mapping approaches. First, most current methods are designed to estimate only a single sea ice parameter, which does not meet the operational need for jointly extracting multiple parameters. Second, due to the complex structures and blurred boundaries of sea ice, prior models have not fully exploited the intrinsic correlations among features. Third, existing multi-task sea ice mapping frameworks typically assign fixed weights to task losses, making it difficult to achieve balanced and coordinated training across tasks.

To address these challenges, this paper proposes an improved semantic segmentation framework, AGL-UNet, with the following main contributions:

(1) A multi-task automated sea ice mapping network is developed to meet practical requirements for simultaneously estimating multiple sea ice parameters, substantially improving operational efficiency.
(2) The ARC block and the GLCM block are introduced to better utilize correlations among multi source inputs and feature associations across different sea ice types, thereby improving the accuracy of sea ice parameter retrieval.
(3) An adaptive loss weighting strategy is proposed, which adjusts task weights based on the gradient norms of shared parameters with respect to each task loss, ensuring more balanced multi-task training.

Following this introduction, Section 2 presents the dataset and methodology, Section 3 describes comparative and ablation experiments, and Section 4 presents the conclusions of this study.

## 2. Materials and Methods

### 2.1. Dataset and Preprocessing

The AI4Arctic dataset, released by the AutoIce Challenge [17], is a labeled dataset widely used in current automated sea ice mapping method development. The dataset covers a total of 532 scenes in the Arctic region monitored by Canadian Ice Service (CIS) and the Danish Meteorological Institute (DMI) from January 2018 to December 2021, all available in NetCDF format.

**Figure 1:**
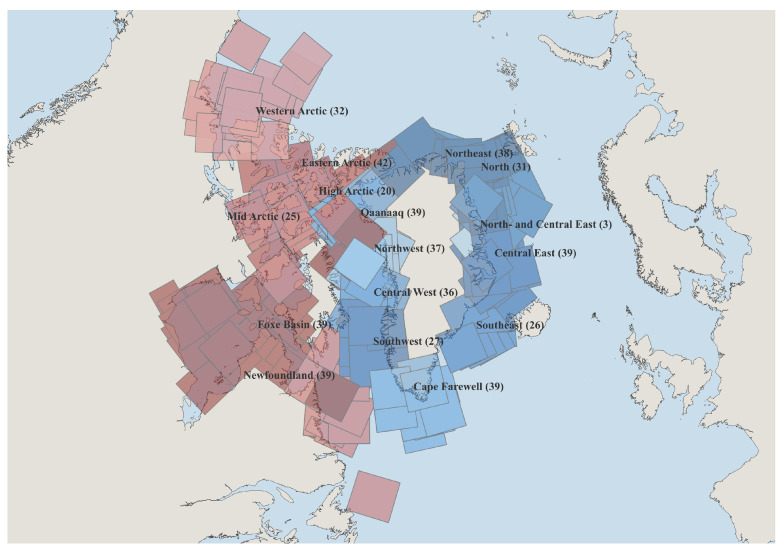
Overview of the spatial distribution of 512 scenes included in the AI4Arctic dataset. The colored polygons represent individual SAR scene footprints, grouped according to the ice charting regions defined by the Canadian Ice Service (CIS, red tones) and the Danish Meteorological Institute (DMI, blue tones). The labels indicate the corresponding region names, and the numbers in parentheses denote the total number of scenes available in each region. The CIS regions include Eastern Arctic, Foxe Basin, High Arctic, Mid Arctic, Newfoundland, and Western Arctic, while the DMI regions include Central East, Southeast, Southwest, Central West, Cape Farewell, Northwest, Qaanaaq, Northeast, North- and Central East, and North.

Specifically, the CIS charting regions include Eastern Arctic, Foxe Basin, High Arctic, Mid Arctic, Newfoundland, and Western Arctic, while the DMI charting regions include Central East, Southeast, Central West, Cape Farewell, Northwest, Qaanaaq, Northeast, North and Central East, and North Greenland waters [18]. These regions jointly cover the major Arctic seas around Canada and Greenland, as illustrated in Figure 1. The dataset spans multiple years and includes observations from both corresponding winter and summer seasons, capturing a wide range of ice conditions and seasonal variability. Among them, 512 scenes are split into the training set and 20 scenes are split into the test set. Each scene integrates multi source observations from AI4Arctic dataset [18], including Sentinel-1 Extra Wide Swath Mode (EW) C-band 5.405 GHz frequency Level 1 SAR data (HH and HV polarizations and incidence angle), a distance-to-land map for all Sentinel-1 pixels (Distance Map), latitude and longitude of subgrid points, AMSR2 brightness temperature channels (e.g., 18.7 and 36.5 GHz in both horizontal and vertical polarizations), ERA5 reanalysis variables (e.g., 10-m wind speed components, 2-m air temperature, total column water vapor, and total column cloud liquid water), and ice maps from the Canadian Ice Service (CIS) and the Danish Meteorological Institute (DMI).

**Figure 2:**
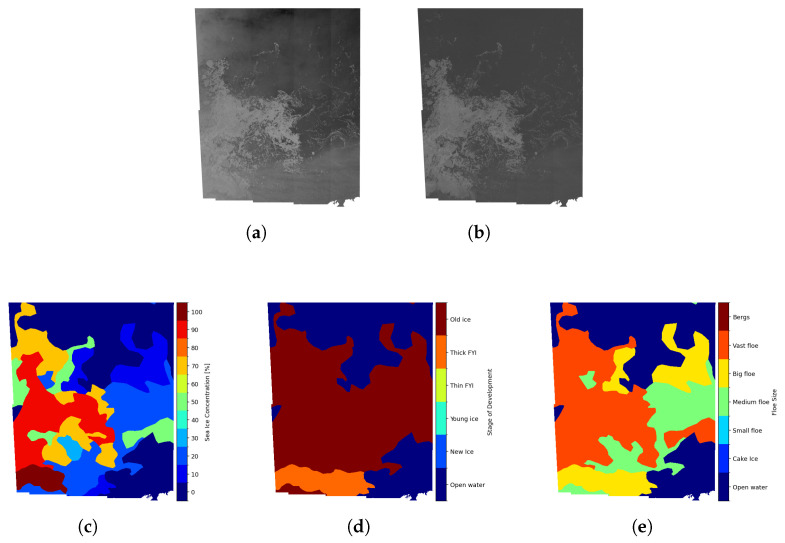
Sentinel-1 SAR scene (ID: 20180821T165051_cis) in the training dataset. (**a**) SAR scene in HH polarization. (**b**) SAR scene in HV polarization. (**c**) SIC map obtained from the corresponding ice chart. (**d**) SOD map obtained from the corresponding ice chart. (**e**) FLOE map obtained from the corresponding ice chart.

The dataset is available in both raw and ready-to-train (RTT) versions. In order to focus on model design and avoid initial data preparation processes, this study adopts the ready-to-train version. This version converts the original ice charts provided by CIS and DMI in WMO SIGRID-3 shapefile format into raster representations stored in NetCDF format, and uniformly resamples SAR images, distance maps, and ice charts to 80 m pixel spacing (approximately 5000 × 5000 pixels). All scenes are normalized to the [−1, 1] range. During the rasterization and resampling process, pixels that cannot be associated with any ice chart polygon (e.g., outside the chart coverage or masked regions) are assigned an ignore value of 255, and the NaN values in the SAR data is uniformly replaced with 2. The SIC (Sea Ice Concentration) represents the percentage of an area covered by sea ice, ranging from 0% (open water) to 100% (fully covered sea ice). The SOD (Stage of Development) represents the stage of sea ice type (a proxy for sea ice thickness) in 6 categories: open water, new ice, young ice, thin FYI (first-year-ice), thick FYI, and old ice. Following the definition of the AI4Arctic dataset [18], the medium FYI category is not included. The FLOE (Floe Size) represents the scale of the ice floe in 7 categories: open water, cake ice (<20 m), small floes (20–100 m), medium floe (100–500 m), big floe (500 m–2 km), vast floe (>2 km) and bergs (including icebergs and glacier ice variants). The SAR scenes of sea ice and sea ice maps of the RTT dataset are shown in Figure 2.

According to [19], the input variables required to construct the model are selected in this study as illustrated in Table 1. These include: SAR variables (HH, HV, and incidence angle), AMSR2 brightness temperature at 18.7 GHz and 36.5 GHz (V and H polarizations), distance map, Latitude and Longitude corresponding to pixels, five ERA5 environment variables, and time channels encoded by SAR acquisition months. Sentinel-1 SAR backscatter signals (HH and HV) and incidence angle are widely used to characterize surface roughness, ice type, and scattering mechanisms. Brightness temperature data acquired by the AMSR2 satellite at 18.7 GHz and 36.5 GHz (H and V polarizations) provide complementary passive microwave information related to ice emissivity properties and thermodynamic state. The distance-to-land map and geographic coordinates (latitude and longitude) aid in distinguishing ice conditions between coastal areas and open-sea regions. ERA5 reanalysis variables (10-m wind components, 2-m air temperature, total column water vapor, and total column cloud liquid water) describe atmospheric forcing related to ice formation, deformation, and melting processes. Temporal encoding using acquisition month captures seasonal variations in sea ice.

Previous studies have demonstrated that removing AMSR2 brightness temperature channels leads to a degradation of 6.4% and 2.2% in SIC and FLOE accuracy on the test set, confirming the importance of passive microwave radiometric information for estimating sea ice concentration and floe size. In contrast, SOD exhibits stronger sensitivity to multi source information: removing AMSR2 inputs, ERA5 atmospheric variables, or spatial-temporal encoding consistently degrades SOD performance, with the largest drop (9.1%) observed when spatial-temporal encoding is excluded. This highlights the critical role of environmental forcing and seasonal evolution in ice type discrimination. These results jointly support the adopted variable configuration in this article [19,20]. In the training phase, we randomly select 17 from 512 training scenes as the validation set, and the remaining 495 are used for training. For testing, 20-scene predefined test set is used. When the model is trained, patches of size (256, 256) are extracted from each scene as training samples.

### 2.2. Methods

**Figure 3:**
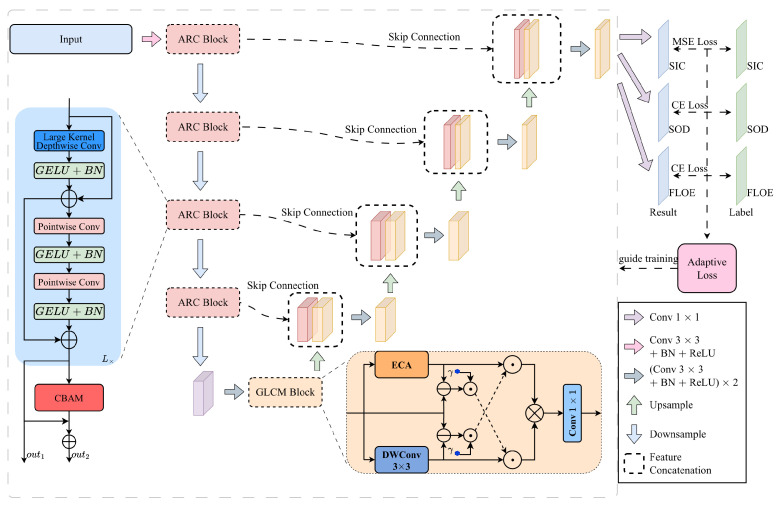
The overall architecture of AGL-UNet.

The overall architecture of AGL-UNet is shown in Figure 3. The encoder stage extracts multi-scale high level semantic features based on the ARC block (Section 2.2.1). Subsequently, an integrated GLCM block (Section 2.2.2) is introduced in the most semantically rich bridging layer to further model the differences between local and non-local features, thereby enhancing semantic representation. The decoder section (Section 2.2.3) adopts the traditional U-Net structure, progressively reconstructing images through skip connections using shallow features from the encoder. Finally, an adaptive multi-task loss weighting strategy based on gradient norms (Section 2.2.4) dynamically balances task learning, thereby promoting efficient training of the overall model.

#### 2.2.1. ARC Block

**Figure 4:**
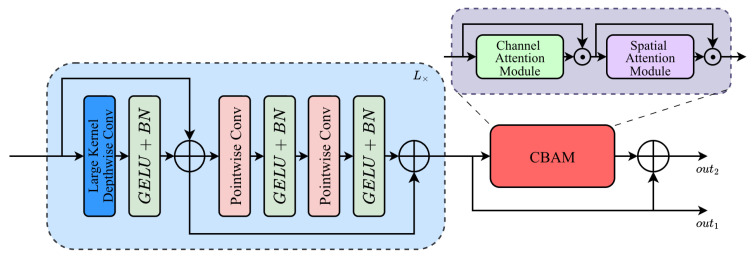
Structure of the ARC module.

Polar sea ice commonly exhibits complex structural deformations, blurred boundaries, and high noise backgrounds, making its semantic representation highly dependent on long range spatial relationships across multiple scales and structural patterns. These characteristics pose substantial challenges for traditional locally constrained convolutional models, whose effective receptive fields are typically much smaller than their theoretical counterparts, thereby limiting their ability to capture global spatial correlations inherent to sea ice morphology. Moreover, noise and weak boundaries cause highly uneven spatial importance distributions, necessitating models capable of both enhanced structural modeling and refined semantic filtering. To address these challenges, we propose the ARC (Adaptive Receptive-field and Context) block, whose overarching objective is to simultaneously expand the effective receptive field, strengthen cross channel structural mapping, and perform adaptive semantic selection. Through this unified design, the ARC block provides a principled mechanism for modeling the intricate and noisy structural properties of polar sea ice while ensuring the extraction of semantically meaningful features. Figure 4 illustrates the ARC block overall architecture.

To capture the cross scale dependencies that characterize sea ice structures, the ARC block begins with a lightweight hybrid convolutional pathway that integrates large-kernel depthwise convolutions with two layers of reverse point convolutions, jointly modeling spatial and channel-wise interactions. This pathway is stacked *L* times, enabling progressive accumulation of spatial context through multi-level transformations, wherein the effective receptive field expands quasi-linearly and inter-channel structural dependencies are repeatedly strengthened. Large-kernel convolutions primarily serve to extend the receptive field, whereas reverse point convolutions provide a low-rank channel mapping mechanism that supports approximations of global structural transformations within a computationally efficient design. Since enhanced structural expressiveness alone does not guarantee optimal semantic discrimination, the block further incorporates the CBAM (Convolutional Block Attention Module) [21], which constructs an adaptive importance weighting function to reweight hybrid-convolution features across spatial and channel dimensions, thereby emphasizing semantically informative regions while suppressing noise. Ultimately, the ARC block outputs two complementary feature types: high resolution detail features for skip connection and contextually enriched, attention filtered deep semantic features for subsequent encoding stage. The overall process can be expressed as

(1) $$\begin{array}{c} X_{1}=X,\;\;\;\;\;\;\;\;\;\;\;\;\;\;\;\;\;\;\;\;\;\;\;\;\;\;\;\;\;\;\;\;\;\;\;\;\;\;\;\;\;\\ \;\;\;\;\;\;\;\;\;\;\;\;\;\;\;\;\;\;Y_{l}=\mathrm{BN}\;\left(\sigma \;\left(\mathrm{DWConv}\left(X_{l};K_{\mathrm{large}}\right)\right)\right)+X_{l},\\ Z_{l}=\mathrm{BN}\;\left(\sigma \;\left(\mathrm{PWConv}\left(Y_{l}\right)\right)\right),\;\;\;\\ \;\;\;\;\;\;\;\;\;\;\;\;\;\;\;\;\;\;\;\;\;\;\;\;\;\;\;\;\;\;\;\;\;X_{l+1}=\mathrm{BN}\;\left(\sigma \;\left(\mathrm{PWConv}\left(Z_{l}\right)\right)\right)+Y_{l},\;l=1,2,\ldots ,L,\\ \omega (X_{L})=A_{c}\left(X_{L}\right)⊙A_{s}\;\left(A_{c}\left(X_{L}\right)⊙X_{L}\right),\;\\ X_{{\mathrm{out}}_{1}}=X_{L},\;\;\;\;\;\;\;\;\;\;\;\;\;\;\;\;\;\;\;\;\;\;\;\;\;\;\;\;\;\;\;\;\;\;\;\;\;\;\;\;\;\;\\ X_{{\mathrm{out}}_{2}}=\omega \left(X_{L}\right)⊙X_{L},\;\;\;\;\;\;\;\;\;\;\;\;\;\;\;\;\;\;\;\;\;\;\;\;\; \end{array}$$

where *X* denotes the input feature map to the ARC block, and $X_{l}$ denotes the output feature map of the *l*-th layer in the hybrid convolutional pathway. DWConv and PWConv refer to the depthwise and pointwise convolutions, respectively, while $K_{\mathrm{large}}$ represents the large convolutional kernel used in the depthwise operation. $Y_{l}$ and $Z_{l}$ are intermediate variables produced by the first and second nonlinear transformations. σ(·) denotes the GELU activation function [22], and BN(·) represents batch normalization. The hybrid convolutional pathway is stacked *L* times to progressively accumulate spatial context and expand the effective receptive field. $A_{c}\left(\cdot \right)$ and $A_{s}\left(\cdot \right)$ correspond to the channel and spatial attention components of the CBAM module, and ω(·) denotes the resulting attention-weighted feature representation. $X_{{\mathrm{out}}_{1}}$ denotes the high-resolution feature map used for the skip connection, whereas $X_{{\mathrm{out}}_{2}}$ denotes the contextually enhanced output feature passed to the next encoder stage, ⊙ is the operation element-wise product. By jointly enhancing receptive-field modeling, structural representation, and adaptive semantic selection, the ARC block significantly improves expressive capacity, feature selectivity, and robustness while maintaining computational efficiency, making it particularly well suited to the complex, noisy, and weakly bounded characteristics of polar sea ice imagery.

#### 2.2.2. GLCM Block

**Figure 5:**
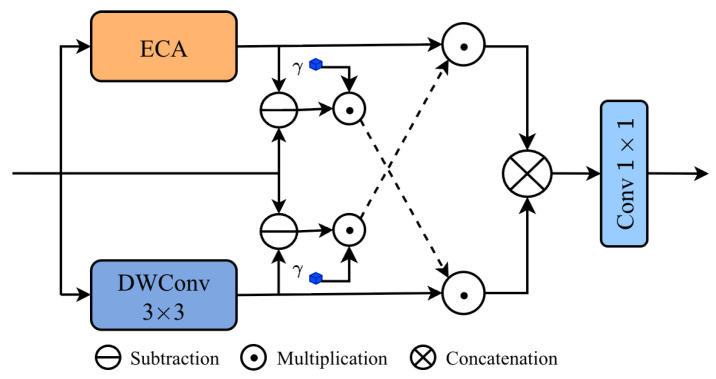
Structure of the GLCM module.

To further enhance the model’s capability to represent and capture informative features, we introduce the Global–Local Cross Modulation (GLCM) block, whose primary aim is to explicitly characterize the feature discrepancies between non-local and local branches within the semantically rich feature space of the bridge layer. As shown in Figure 5, given an input feature *X*, the non-local channel feature $X_{E}$ is derived using Efficient Channel Attention (ECA) [23], while the local spatial feature $X_{D}$ is extracted through a light weight local aggregation module based on a DWConv3×3 operator. ECA performs channel-level attention refinement through light weight cross-channel interactions without dimensionality reduction, thereby enhancing key semantic channels and suppressing noisy ones while effectively capturing global channel dependencies. In parallel, the DWConv3×3 branch aggregates local spatial context to obtain fine-grained spatial structures, including edges and textures, and further strengthens trunk features via residual enhancement. Collectively, these two complementary branches provide a comprehensive framework for modeling both global and local characteristics of the input feature space.

Building upon the features extracted from the two branches, we compute the difference between the input feature and each branch output, introducing a learnable scaling factor γ to adaptively modulate these discrepancy. By matching the discrepancy information derived from the ECA branch with the feature representation of the DWConv3×3 branch, a gating mechanism is established to modulate local spatial features; similarly, matching the discrepancy from the DWConv3×3 branch with the ECA-derived features constructs a complementary gating mechanism for non-local feature modulation. The outputs of the two modulated branches are subsequently concatenated along the channel dimension and fused through a 1×1 convolution to generate the final representation. For an input feature *X*, this overall process can be expressed as

(2) $$\begin{array}{c} X_{E},\;X_{D}=F_{\mathrm{ECA}}\left(X\right),\;F_{\mathrm{DWConv}3\times 3}\left(X\right),\\ X_{E}^{\mathrm{sub}},\;X_{D}^{\mathrm{sub}}=\gamma ⊙\left(X-X_{E}\right),\;\gamma ⊙\left(X-X_{D}\right),\\ X_{\mathrm{out}}^{E},\;X_{\mathrm{out}}^{D}=X_{E}⊙X_{D}^{\mathrm{sub}},\;X_{D}⊙X_{E}^{\mathrm{sub}},\;\;\;\;\;\;\;\;\;\;\;\\ X_{\mathrm{out}}={\mathrm{Conv}}_{1\times 1}\;\left(X_{\mathrm{out}}^{E}⊗X_{\mathrm{out}}^{D}\right),\;\; \end{array}$$

where $X_{E}^{\mathrm{sub}}$ and $X_{D}^{\mathrm{sub}}$ indicate the discrepancy information of the ECA branch and the DWConv3×3 branch, respectively. γ is the learnable scaling factor. ⊗ and ⊙ denote the operations of channel concatenation and element-wise multiplication, respectively. $X_{\mathrm{out}}$ is the final output of the GLCM block.

#### 2.2.3. U-Net Decoder

The decoder stage follows the conventional U-Net architecture [24]. Along the encoding pathway, the multi-source input features undergo progressive spatial compression as higher-level semantic representations are extracted. Because accurate delineation of heterogeneous sea-ice types is essential, the symmetric U-Net structure and its skip-connection mechanism play a critical role in mitigating the loss of spatial detail introduced during encoding. These skip connections bridge the gap between semantic abstraction and spatial precision, effectively restoring fine-grained information that would otherwise be lost.

The high-level semantic features generated by the GLCM module are forwarded to the decoder, where spatial resolution is recovered through bilinear interpolation—a stable, parameter-free upsampling strategy that avoids checkerboard artifacts and preserves geometric consistency with encoder features. By injecting high-resolution information from earlier encoding stages, the skip connections enhance boundary localization and facilitate multi-scale feature aggregation. This fusion of deep semantic cues with shallow spatial details substantially improves recognition performance across diverse spatial scales.The segmentation outputs achieve both semantic coherence and spatial fidelity, thereby meeting the rigorous requirements of complex sea ice parameter analysis.

#### 2.2.4. Adaptive Loss Weighting Method

Multitask learning aims to jointly optimize multiple related tasks by leveraging shared parameters, and its overarching goal is to achieve balanced improvements across tasks. However, conventional approaches typically adopt a linearly weighted average of task losses, a strategy that often fails to account for heterogeneous task characteristics and therefore cannot maintain an appropriate optimization balance. To address this limitation, prior work [25] proposes assigning weights based on the inverse of each task’s stop-gradient loss, formulated as

(3) $$L=\sum_{i=1}^{n}\frac{L_{i}}{L_{i}^{\left(\mathrm{sg}\right)}}$$

where *n* is the total number of tasks, L denotes the overall multi-task loss, $L_{i}$ is the loss of the *i*th task, and $L_{i}^{\left(\mathrm{sg}\right)}$ denotes its stop-gradient form. Since loss functions measure the deviation between the model state and its target, and gradient descent minimizes this deviation by searching for points with vanishing gradients, the magnitude of the gradient offers a natural and theoretically grounded signal for regulating multi-task optimization. Building on this observation, a gradient-normalized weighting strategy can be constructed by scaling each task loss with the inverse L2 norm of its gradient with respect to the shared parameters θ:

(4) $$L=\sum_{i=1}^{n}\frac{L_{i}}{{∥\nabla_{\theta }L_{i}∥}^{\left(\mathrm{sg}\right)}}$$

Although this formulation provides dynamic and scale-invariant regulation, empirical results reveal that the SIC regression task exhibits substantially larger gradient magnitudes than the SOD and FLOE classification tasks. As a consequence, the induced gradient-normalized weight for SIC becomes excessively small, contradicting its practical importance as SIC serves as a guiding signal for sea ice type classification [19]. To reconcile this imbalance, the weighting strategy was refined along three steps. First, the gradient magnitudes across tasks were averaged to equalize their contributions to shared-parameter updates. Second, a lower bound was imposed on the SIC weight, ensuring that it remains no less than one-third of the weights assigned to the SOD and FLOE tasks. Third, all weights were normalized such that their sum equals the number of tasks, thereby promoting stable joint optimization. The resulting formulations are

(5) $$\begin{array}{cc} & {\tilde{w}}_{i}=\frac{\frac{1}{n}\sum_{j=1}^{n}{∥\nabla_{W}L_{j}∥}^{\left(\mathrm{sg}\right)}}{{∥\nabla_{W}L_{i}∥}^{\left(\mathrm{sg}\right)}},\\ & \;\;\;\;\;\;\;\;\;\;\;\;\;\;\;\;\;\;\;\;\;\;\;\;\;\;{\tilde{w}}_{\mathrm{SIC}}=\max\;\left({\tilde{w}}_{\mathrm{SIC}},\frac{1}{3}\min\;\left({\tilde{w}}_{\mathrm{SOD}},{\tilde{w}}_{\mathrm{FLOE}}\right)\right),\\ & w_{i}={\tilde{w}}_{i}\frac{n}{\sum_{j=1}^{n}{\tilde{w}}_{j}},\;\;\;\;\;\;\;\;\;\;\;\\ & L=\sum_{i=1}^{n}w_{i}\;L_{i}.\;\;\;\;\;\;\;\;\;\;\;\;\;\; \end{array}$$

where *W* denotes the parameters of the final decoder block shared across all tasks, and $\tilde{w}$ and *w* represent the weights before and after normalization, respectively.

Together, these refinements yield a more robust and interpretable weighting mechanism that preserves gradient comparability, prevents the regression task from being underweighted, and enhances the overall stability and effectiveness of multi-task learning. The lower bound imposed on SIC task weights is not introduced as a temporary heuristic but as a stability constraint to prevent task starvation under strong gradient competition during multi-task optimization. In our multi-task setting (SIC, SOD, and FLOE), the loss value of the SIC MSE loss function is approximately three times that of the other two classification tasks in each epoch in this study, providing a natural reference scale for maintaining balanced task participation. The adaptive weighting mechanism is primarily driven by gradient normalization, with the lower bound activated only during extreme imbalances to stabilize training. In practice, we observe that training behavior remains insensitive to minor variations in this threshold, as the constraint primarily impacts the rare instances of severe gradient dominance. More generally, this design principle can be extended to other multi-task learning problems by making the minimum weight for N tasks proportional to the ratio of the maximum loss to the minimum loss across multiple tasks. This provides a simple and scalable mechanism that avoids task suppression while preserving the flexibility of adaptive weighting strategies.

## 3. Experiments

### 3.1. Experimental Details

The training specifications are summarized in Table 2, which lists the optimal hyperparameters obtained from experiments. A cosine annealing warm-restart schedule [26] is employed to modulate the learning rate, where the rate gradually decreases to a predefined minimum following a cosine function and then restarts to its initial value every 20 iterations. This scheduling strategy enables the optimizer to explore different regions of the loss landscape and reduces the risk of convergence to suboptimal local minima. Each epoch consists of 500 iterations to provide sufficient stochastic sampling of patches extracted from the training scenes. During training, mean square error (MSE) is used as the loss function for the SIC regression task, while cross-entropy loss is applied to the SOD and FLOE classification tasks. At the end of each epoch, a combined score is computed on the validation set using the metrics listed in Table 3, and the model parameters are updated whenever an improved combined score is observed. After training, the best-performing model is used to generate predictions for all scenes in the test set. All experiments are performed on an NVIDIA RTX 4090 GPU with 24 GB of memory and a system equipped with 120 GB RAM, using the PyTorch 1.12 library.

### 3.2. Evaluation Metrics

The evaluation metrics are defined in accordance with the requirements in the AutoIce Challenge. SIC results are assessed by calculating the $R^{2}$ score. $R^{2}$ score is defined as

(6) $$R^{2}=1-\frac{\sum_{i=1}^{n}{\left(y_{i}-{\hat{y}}_{i}\right)}^{2}}{\sum_{i=1}^{n}{\left(y_{i}-\bar{y}\right)}^{2}}$$

where *n* is the number of pixels, and $y_{i}$ and ${\hat{y}}_{i}$ are the ground truth and estimated SIC of the *i*th pixel, respectively. $\bar{y}$ is the mean SIC across all ground-truth observations. Both SOD and FLOE maps are assessed using the F1 score, which is defined as

(7) $$F1=2\times \frac{\mathrm{Precision}\times \mathrm{Recall}}{\mathrm{Precision}+\mathrm{Recall}}$$

where Precision=(TP/(TP+FP)), Recall=(TP/(TP+FN)), and TP, FP, and FN denote the true positives, false positives, and false negatives of all valid pixels in the test set, respectively.

The three sea ice parameter scores will be combined into a single final score, as defined in the weighting scheme shown in Table 3.

### 3.3. Experimental Results

To evaluate the performance of the proposed AGL-UNet, we compared it with several recently developed models on the AI4Arctic dataset, including [19,27,28]. The model was trained several times to ensure the robustness and reliability of the results. During each training run, model performance is monitored throughout convergence, and the checkpoint achieving the highest validation score is selected. The corresponding weights are then used to evaluate the model on the test set to obtain the final performance metrics.

**Figure 6:**
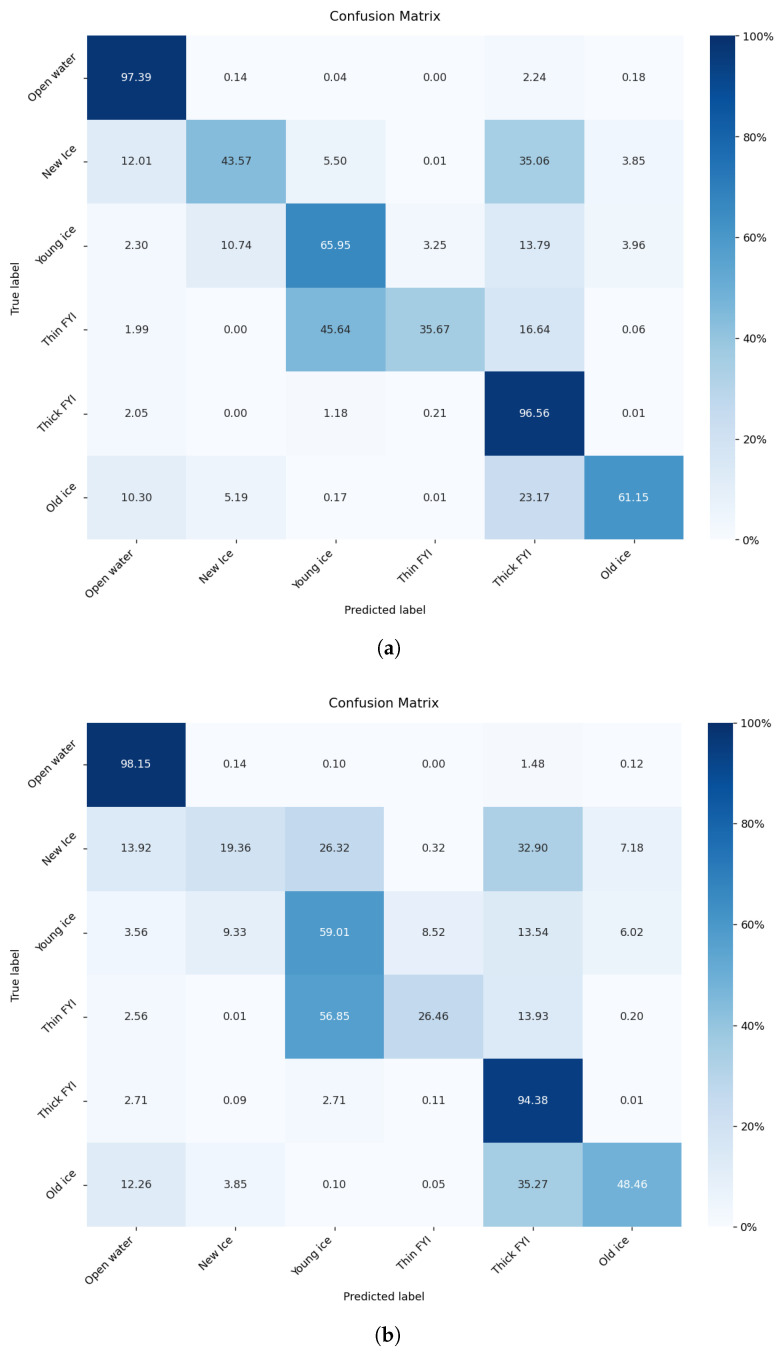
Confusion matrices of SOD. (**a**) SOD confusion matrix of AGL-UNet. (**b**) SOD confusion matrix of the top performing method in the AutoIce competition.

Table 4 summarizes the performance of the proposed model compared with previously reported models, with all results rounded to two decimal places. All competing models, including ours, are trained on the RTT version of the dataset. As shown in the table, the proposed model achieved an improvement over the current state-of-the-art methods, with a combined score of 87.63. Specifically, the SIC $R^{2}$ score is 90.45, the SOD F1 score is 90.05, and the FLOE F1 score is 77.14. These results demonstrate that the proposed model effectively balances the three tasks, with SOD exhibiting the strongest classification performance in the multi-task setting. The confusion matrices in Figure 6 further show that prediction SOD is more concentrated along the diagonal, indicating improved class separability. The original class imbalance issues in the dataset have also been mitigated, contributing to more stable learning for SOD within the multi-task framework.

**Figure 7:**
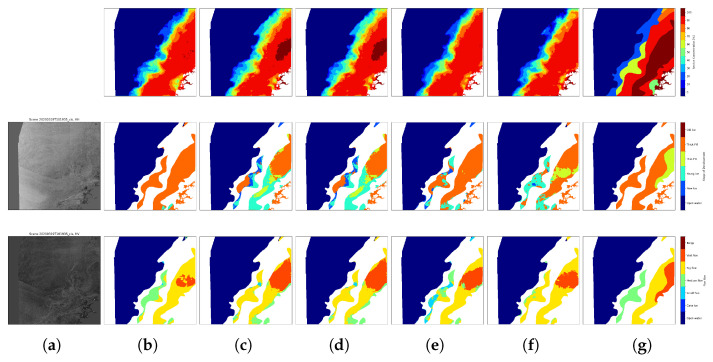
Sea ice mapping results obtained from a SAR scene (ID: 20200319T01935_cis) in the testing data using models trained with different configurations. The three rows are SIC, SOD, and FLOE for the scene. (**a**) SAR scenes. (**b**) Predictive results of the proposed model. (**c**) Predictive results of the proposed model removing ARC blocks. (**d**) Predictive results of the proposed model removing GLCM block. (**e**) Predictive results of the proposed model removing CBAM block. (**f**) Predictive results of the proposed model removing adaptive weighting method. (**g**) Ice-chart-derived labels.

**Figure 8:**
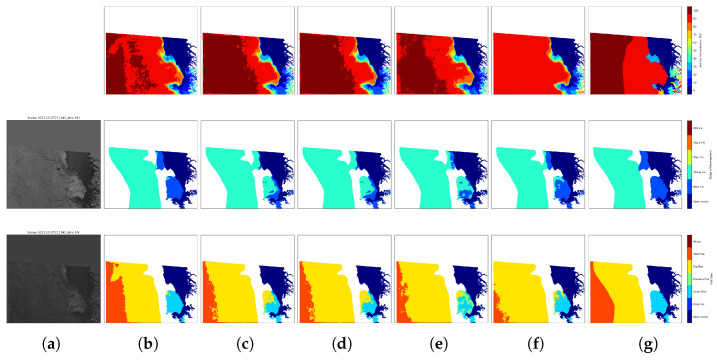
Sea ice mapping results obtained from a SAR scene (ID: 20211212T211242_dmi) in the testing data using models trained with different configurations, the three rows are SIC, SOD, and FLOE for the scene. (**a**) SAR scenes. (**b**) Predictive results of the proposed model. (**c**) Predictive results of the proposed model removing ARC blocks. (**d**) Predictive results of the proposed model removing GLCM block. (**e**) Predictive results of the proposed model removing CBAM block. (**f**) Predictive results of the proposed model removing adaptive weighting method. (**g**) Icechart-derived labels.

Visualization results for two representative test scenes are presented in Figure 7 and Figure 8. These scenes were selected to cover as many classes as possible across the three tasks, allowing for a comprehensive qualitative assessment. In both figures, the second column shows the model predictions for SIC, SOD, and FLOE, while the last column presents the corresponding ground-truth labels. The predictions exhibit strong visual alignment with the ground truth. For SIC, although small errors persist, discrepancies are limited to specific regions. For SOD, the majority of regions are classified correctly, with misclassifications mainly occurring surrounding thin first year ice, typically concentrated along boundary zones. FLOE classification, which is inherently more challenging, also shows high accuracy across most regions, further supporting the effectiveness of the proposed model.

### 3.4. Ablation Experiments

To assess the effectiveness and necessity of the ARC block, the GLCM block, and the Adaptive Loss Weighting Method within the proposed AGL-UNet for multi-task sea ice mapping, a series of ablation studies were performed by progressively removing individual components and examining the resulting performance changes. Each modified model was trained several times to ensure the robustness of the reported results, with the removed modules and corresponding quantitative outcomes summarized in Table 5. The trained variants are denoted as Model 1 through Model 5, and the final score for each model represents the average performance across several runs under identical experimental configurations.

The comparative performance of the different model variants is shown in Table 5. Model 1 corresponds to the full proposed configuration described in Section 3.1 and Table 2. Model 2 removes the ARC block, Model 3 removes the GLCM block, and Model 4 removes the CBAM block to verify its contribution within the ARC block. Model 5 replaces the proposed adaptive loss weighting strategy with a fixed linear weighting scheme (1:3:3) in [19]. To ensure fair comparison, all models were trained and evaluated using identical datasets and training protocols. As shown in Table 5, removing the ARC block in Model 2 leads to decreases 0.35% in SOD and 0.43% in FLOE compared with Model 1. Model 3 confirms the effectiveness of the GLCM block, highlighting the importance of GLCM for improving SOD classification accuracy, removing the GLCM block in Model3 leads to decreases 4.2% in SOD and 4.61% in FLOE compared with Model 1. Model 4 demonstrates the necessity of CBAM block for enhancing the model’s feature selection capabilities, removing the CBAM block in Model 2 leads to decreases 2.8% in SOD compared with Model 1. The contribution of the adaptive loss weighting method is illustrated by Model 5: compared with Model 1, the SOD, and FLOE scores respectively decrease by 1.32% and 0.8%, reflecting the importance of dynamic weighting in balancing task contributions. Visualization examples for Models 2–5 are presented in Columns c-f of Figure 7 and Figure 8. The matching degree between the visualized results and the truth values of these models is significantly lower than that of Model 1.

### 3.5. Discussion

Although the proposed model exhibits a slight decrease in SIC accuracy compared with the baseline, this behavior aligns with the inherent trade-off commonly observed in multi-task learning, where shared representations are optimized to balance multiple objectives rather than a single metric. In practical applications, the observed SIC variations do not alter the overall spatial patterns or concentration states, as illustrated in Figure 7 and Figure 8, where row 1 and columns (b) and (f) correspond to the full proposed model and the variant without the adaptive weighting method, respectively. In addition, Column SIC in Table 5 shows that the accuracy remains nearly unchanged. Improvements in SOD and FLOE provide additional physically meaningful information that SIC alone cannot capture, such as ice type evolution and floe size distribution. These variables are highly relevant for navigation safety, ice dynamics analysis, and comprehensive sea ice monitoring. Furthermore, the increase in the combined score indicates an overall improvement in multi-task performance rather than a shift toward single task optimization. This demonstrates that the proposed model achieves a more balanced representation of complementary sea ice parameters, where enhancements in the categorical structure (SOD and FLOE) compensate for a slight regression trade-off in SIC, thereby enhancing the overall informational content and practical utility.

The experimental evaluation in this study is based on the AI4Arctic RTT dataset, which is geographically focused on waters around Canada and Greenland and spans the period from 2018 to 2021. Although this dataset covers diverse regions and all seasons with significant variability, the generalizability of the model to other regions and different sensor configurations has not been explicitly validated. Nevertheless, the input variables employ SAR backscatter, passive microwave brightness temperature, and ERA5 reanalysis variables exhibit physical consistency across regions and seasons, providing a foundation for transferable feature learning. Furthermore, the multi scale architecture and multi-task learning framework aim to capture generic structural and physical characteristics of sea ice rather than specific region patterns, suggesting potential generalization beyond the current dataset. Future work will further investigate cross-region test, seasonal split experiments, and multi sensors transfer studies to systematically assess robustness and generalization under broader scenarios.

## 4. Conclusions

The proposed AGL-UNet extends the U-Net architecture through the integration of ARC blocks, a GLCM block, and an adaptive loss weighting strategy, achieving strong performance in multi-task sea ice mapping. All results are computed as averages over the entire test set in the RTT dataset. The test samples include all four seasons and span diverse regions in Canadian and Greenland waters, thereby including both stable winter ice conditions and highly variable summer marginal ice zones and reducing potential geographical bias. This diverse spatial-temporal coverage ensures that the reported results reflect overall generalization performance rather than being biased toward a specific region or season. Compared with existing approaches based on U-Net [19], the proposed model yields improvements of 2.85% and 3.44% in SOD and FLOE F1 scores, respectively, while exhibiting only a marginal decrease in SIC accuracy. The combined score increases by 1.33%, indicating that the model effectively balances multi-task learning objectives. Since all models are evaluated on exactly the same test set using identical metrics, the observed performance differences directly reflect the effectiveness of the proposed approach rather than dataset or evaluation variability. Moreover, the improvement in the combined score demonstrates enhanced overall multi-task capability instead of isolated metric fluctuations. Ablation experiments further confirm that the ARC and GLCM blocks substantially enhance the model’s ability to identify marginal sea ice features and small ice floes. The adaptive loss weighting method also contributes to improved SOD and FLOE performance by equalizing training dynamics across tasks, thereby boosting the combined score. Future work may explore more advanced multi-task balancing strategies to address the inherent long-tail distribution in polar sea ice datasets. Additionally, incorporating richer observational sources (e.g., optical and infrared data from sensors MODIS and MERSI-II) offers a promising direction for further enhancing model robustness and generalization.

## Figures and Tables

**Table 1:** Observation groups used as input to the models.

Obervation Group	Variable Description	Number of Channels
SAR	HH, HV, incidence angle	3
AMSR2	Dual-polarized AMSR2 brightness temperature data in 18.7 and 36.5 GHz	4
ERA5	10-m wind speed, 2-m air temperature, total column water vapor, total column cloud liquid water	5
Location, time	Latitude/longitude of each pixel, distance map and scene acquisition month	4

**Table 2:** Specifications of training the proposed model with the highest combined score, including hyperparameter values, learning algorithms, and loss functions.

Optimizer	Stochastic Gradient Descent with Momentum (SGDM)
Learning rate	0.001
Weight decay	0.01
Scheduler	Cosine annealing with warm restarts
Batch size	16
Number of iterations per epoch	500
Total epoch	200
Number of epochs for the first restart	20
Downscaling ratio	10
Data augmentation	Rotation, flip, random scale, CutMix
Patch size	256
ARC Block Lengths	1,1,3,3
ARC Block Kernel Size	3,3,5,5
Loss functions	Mean square error loss for SIC, cross entropy loss for SOD and FLOE

**Table 3:** Evaluation metrics and respective weights in the final score.

Sea Ice Parameter	Metric (%)	Weight in Combined Score
SIC	$R^{2}$	2/5
SOD	F1	2/5
FLOE	F1	1/5

**Table 4:** Results of comparative experiments on AI4Arctic RTT dataset. The results in bold show the best performance.

Method	SIC $R^{2}$(%)	SOD F1(%)	FLOE F1(%)	Combined Score (%)
[19]	91.7	87.2	73.7	86.3
[27]	91.33	89.35	75.11	87.3
[28]	89.95	90.03	77.69	87.53
AGL-UNet	90.45	90.05	77.14	87.63

**Table 5:** Results of ablation experiments on AI4Arctic RTT dataset. The results in bold show the best performance.

Model Number	Modifications Compared to Model 1	SIC $R^{2}$ (%)	SOD F1 (%)	FLOE F1 (%)	Combined Score (%)
1	N/A (full model)	90.45	90.05	77.14	87.63
2	Remove ARC block	90.68	89.7	76.71	87.49
3	Remove GLCM block	90.37	85.85	72.53	84.99
4	Remove CBAM block	90.93	87.25	77.51	86.78
5	Remove adaptive loss weighting method	90.72	88.73	76.34	87.05

## Data Availability

The AI4Arctic dataset is available from https://doi.org/10.11583/DTU.21284967 (accessed on 9 October 2025).
